# Functional connectivity associated with attention networks differs among subgroups of fibromyalgia patients: an observational case–control study

**DOI:** 10.1038/s41598-024-60993-9

**Published:** 2024-05-03

**Authors:** Tomohiko Aoe, Ryoko Kawanaka, Fumio Ohsone, Akira Hara, Tokuzo Yokokawa

**Affiliations:** 1 Pain Center, Chiba Medical Center, Teikyo University, 3426-3 Anesaki, Ichihara, Chiba 299-0111 Japan; 2 Department of Anesthesiology, Chiba Medical Center, Teikyo University, 3426-3 Anesaki, Ichihara, Chiba 299-0111 Japan; 3 Department of Radiology, Chiba Medical Center, Teikyo University, 3426-3 Anesaki, Ichihara , Chiba 299-0111 Japan

**Keywords:** Neuropathic pain, Fibromyalgia

## Abstract

Fibromyalgia is a heterogenous chronic pain disorder diagnosed by symptom-based criteria. The aim of this study was to clarify different pathophysiological characteristics between subgroups of patients with fibromyalgia. We identified subgroups with distinct pain thresholds: those with a low pressure pain threshold (PL; 16 patients) and those with a normal pressure pain threshold (PN; 15 patients). Both groups experienced severe pain. We performed resting-state functional MRI analysis and detected 11 functional connectivity pairs among all 164 ROIs with distinct difference between the two groups (*p* < 0.001). The most distinctive one was that the PN group had significantly higher functional connectivity between the secondary somatosensory area and the dorsal attention network (*p* < 0.0001). Then, we investigated the transmission pathway of pain stimuli. Functional connectivity of the thalamus to the insular cortex was significantly higher in the PL group (*p* < 0.01 – 0.05). These results suggest that endogenous pain driven by top-down signals via the dorsal attention network may contribute to pain sensation in a subgroup of fibromyalgia patients with a normal pain threshold. Besides, external pain driven by bottom-up signals via the spinothalamic tract may contribute to pain sensations in another group of patients with a low pain threshold.

Trial registration: UMIN000037712.

## Introduction

Fibromyalgia is a chronic pain disorder caused by unknown factors and characterized by widespread pain with other symptoms, including fatigue, sleep disorder, and anxiety^1^. Fibromyalgia affects four million adults in the USA (Centers for Disease Control and Prevention, https://www.cdc.gov/arthritis/types/fibromyalgia.htm). The prevalence of chronic pain with fibromyalgia was reported to be 5.4% in the UK^2^ and 2.1% in Japan^3^. The most commonly used diagnostic criteria was published by the American College of Rheumatology (ACR); these criteria evaluate whether or not tenderness is felt in 18 different parts of the body^4^. In contrast, the 2010 and 2016 ACR criteria provide a diagnosis based on subjective pain perception and other symptoms^1^^,^^5^. Objective laboratory data, including blood tests and diagnostic imaging, are not considered as diagnostic criteria. These symptom-based criteria, and the lack of objectivity, may cause confusion for patients and physicians when managing fibromyalgia.

Fibromyalgia may have a variety of etiologies^6^, including immune disorders (rheumatoid arthritis^7^, viral infections^8^, and vaccination^9^) and physical factors (trauma and surgery)^10^. Tissue damage and inflammation may cause persistent peripheral nociceptive pain input^11^ while small fiber neuropathy may cause neuropathic pain^12^. Psychological and social emotional factors may also be involved^13^. Considering these associated factors, there may not be a single integrated pathophysiological state that can explain the condition of all patients with fibromyalgia.

In addition to pain, most patients experience symptoms related to the central nervous system (CNS), including fatigue, sleep disorders, cognitive dysfunction, anxiety and depression^14^. Fibromyalgia is considered as a nociplastic pain disorder^15^. Changes in sensory processing and pain modulation in the CNS may contribute to widespread pain. Imaging studies have observed a variety of neural communication changes^6^. Decreased gray matter volumes in the prefrontal cortex, the amygdala, and the anterior cingulate cortex have also been observed in patients with fibromyalgia^16^. Therefore, it is thought that changes may occur both functionally and structurally. Resting-state functional magnetic resonance imaging (rs-fMRI) analysis can non-invasively evaluate the degree of coupling of neural activity between any two brain regions. Patients with fibromyalgia exhibit changes in the neural connections between the default mode network (DMN)^17^, salience network^18^, somatosensory cortex^19^, insular cortex^20^, amygdala^21^, periaqueductal gray (PAG)^22^, the dorsal attention network^23^, and other parts of the brain. The insular cortex and secondary somatosensory cortex receives sensory signals from the spinothalamic tracts^24^^,^^25^. The descending pain modulatory system, including the PAG in the brainstem, controls the threshold for sensory input^26^. It is unclear whether these changes are the results or causes of intense pain sensation^17^.

We have observed that some patients with fibromyalgia feel widespread ongoing pain due to an extremely low pain threshold, while others complain of spontaneous widespread ongoing pain but have a normal pain threshold. Here, we used rs-fMRI to investigate differences in the neural connections of these two patient groups. We compared functional connectivity (FC) between different regions-of-interest (ROIs) and identified several connections that differed significantly between the two groups. In addition, we discuss differences in pathophysiological characteristics between different subgroups of patients with fibromyalgia and suitable treatments.

## Results

**Figure 1:**
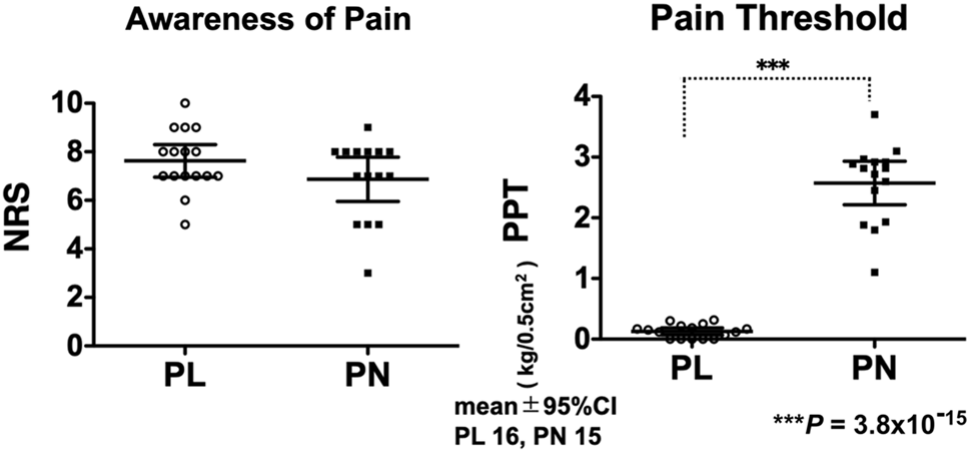
A significant difference between the two groups for pain threshold**.** There were no significant differences between the two groups in terms of the degree of pain (numeric rating scale score, NRS; PL; 7.6 (± 1.3) vs. PN; 6.9 (± 1.6), *p* = 0.158)**.** However, there was a significant difference in pressure pain threshold, PPT, (PL; 0.13 (± 0.11) vs. PN; 2.57 (± 0.65), *p* = 3.80 × 10^−15^) between the PL and PN groups. The pain threshold in the PN group was equivalent to that in subjects with no pain, as previously determined in a previous study (25 females, age; 42.6 (± 7.3), pressure pain threshold; 2.44 (± 0.75), Student’s t test; *p* = 0.572 vs. PN, *J. Clin. Med. *2022, 11(19), 5587). Graphs represent mean and 95% CI values for each group and each dot represents a different patient.

During the study period, 31 patients who met the 2016 ACR diagnostic criteria were included. We aimed to recruit 17 individuals in each group, but the target number was not reached (Table 1). Based on pressure pain threshold (PPT) values, 16 patients (2 males and 14 females) were assigned to the PL group (low pain threshold) and 15 patients (1 male and 14 females) were assigned to the PN group (normal pain threshold). There were no significant differences between the two groups with regards to the degree of pain (numeric rating scale, NRS score; PL: 7.6 ± 1.3 vs. PN: 6.9 ± 1.6; *p* = 0.158) or pain catastrophizing scale (PCS) score (PL: 37.5 ± 8.2 vs. PN: 38.5 ± 6.5; *p* = 0.711; one datapoint was missing from each group). The 2016 ACR scores were significantly higher in the PL group (PL: 23.3 ± 4.9 vs. PN: 19.0 ± 3.7; *p* < 0.01). The widespread pain index (WPI) scores were significantly higher in the PL group (PL: 14.9 ± 3.3 vs. PN: 10.9 ± 2.8; *p* < 0.01). The symptom severity scale (SSS) scores were not significantly different when compared between the two groups (PL: 8.4 ± 2.1 vs. PN: 7.9 ± 1.9; *p* = 0.429). There was a significant difference between the two groups with regards to pain threshold (PPT; PL: 0.13 ± 0.11 vs. PN: 2.57 ± 0.65; *p* = 3.80 × 10^−15^; Fig. 1; Table 1). The pain threshold in the PN group was equivalent to that in subjects with no pain who were investigated previously in another study (25 females; age: 42.6 ± 7.3; PPT: 2.44 ± 0.75; Student’s t test; *p* = 0.572 vs. PN)^27^.

**Table 1:** Patient characteristics.

	PL (mean ± SD)	PN (mean ± SD)	*p* value for Student’s t test
Age	41.6 ± 12.7	43.5 ± 8.94	0.647
Sex	Male 2, female 14	Male 1, female 14	
NRS	7.63 ± 1.26	6.87 ± 1.64	0.158
PPT kg/0.5cm^2^	0.13 ± 0.11	2.57 ± 0.65	3.80 × 10^−15^
ACR2016 (0–31)	23.3 ± 4.85	19.0 ± 3.72	9.87 × 10^−3^
WPI (0–19)	14.9 ± 3.34	10.9 ± 2.84	1.43 × 10^−3^
SSS (0–12)	8.44 ± 2.10	7.87 ± 1.85	0.429
PCS (0–52)	37.5 ± 8.18	38.5 ± 6.53	0.711

Based on pressure pain threshold (PPT) values (see Methods section), 16 patients were assigned to the PL group (low pain threshold) and 15 patients were assigned to the PN group (normal pain threshold). The patients of both groups complained of similar widespread pain and were diagnosed with fibromyalgia based on the American College of Rheumatology 2016 criteria; there was a marked difference in their thresholds to pain. The numbers represent the mean (SD). Values in the PL group and PN group were analyzed by student’s *t* test.

*NRS* Numeric Rating Scale, *PPT* pressure pain threshold, *ACR* american college of rheumatology, *WPI* widespread pain index, *SSS* symptom severity scale, *PCS* pain catastrophizing scale.

**Figure 2:**
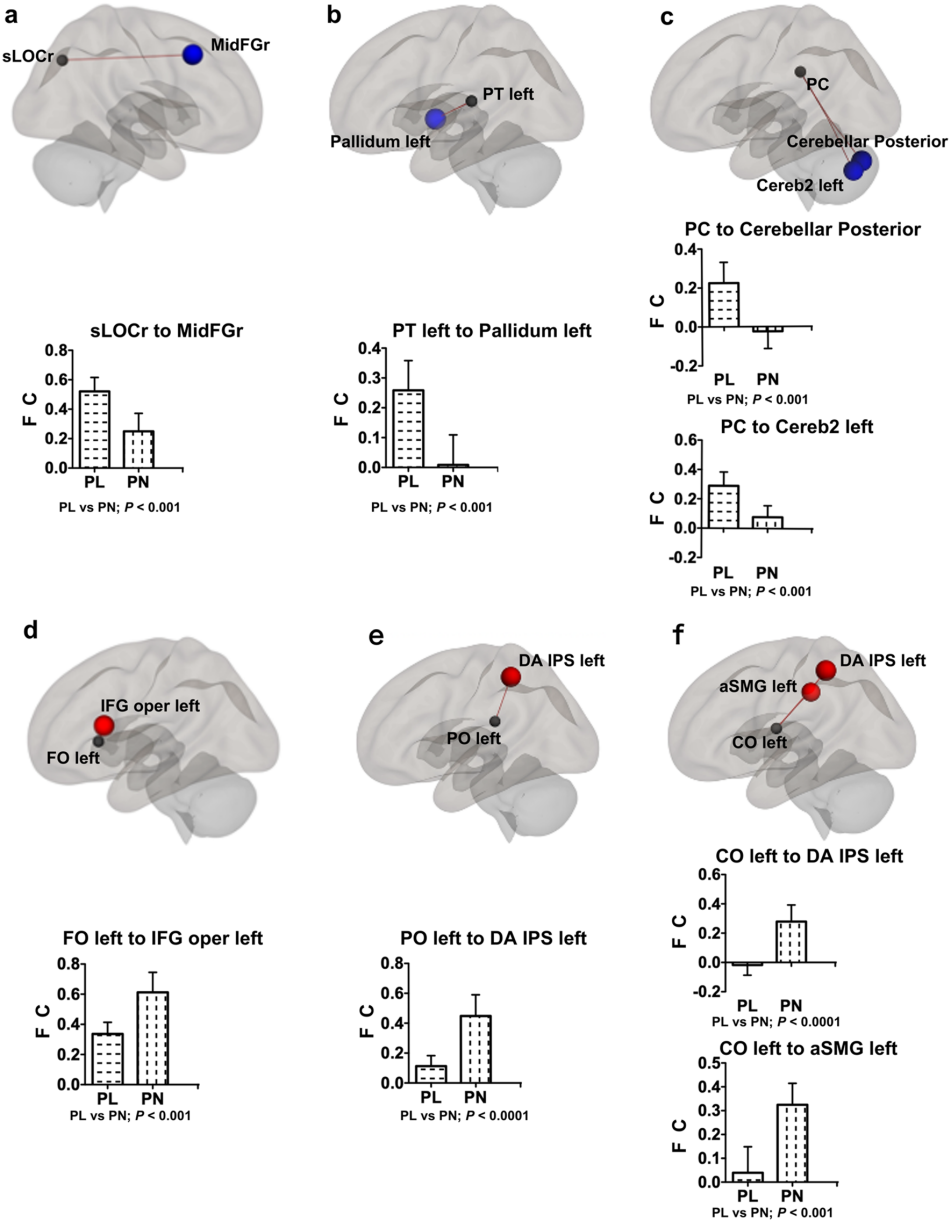
Connected pairs with significantly different functional connectivity between the two patient groups. (**a**–**c**) The functional connectivity in the PL group were significantly larger than those of the PN group (*p* < 0.001). (**d**–**f**) The functional connectivity in the PN group were significantly larger than those of the PL group (*p* < 0.001). Graphs represent mean and 95% CI values for each group. *FC* functional connectivity, *sLOCr* superior division of lateral occipital cortex right, *MidFGr* middle frontal gyrus right, *PT left* planum temporale left, *PC* posterior division of cingulate gyrus, *Cereb2 left* cerebellum crus2 left, *FO left* frontal operculum cortex left, *IFG oper left* pars opercularis of inferior frontal gyrus left, *PO left* parietal operculum cortex left, *DA IPS left* intraparietal sulcus in dorsal attention network left, *CO left* central opercular cortex left, *aSMG left* anterior supramarginal gyrus left.

We investigated ROI-to-ROI FC to identify distinct pathogenic characteristics between the two groups. Between-group analyses of 13366 connections across 164 ROIs detected 11 connected pairs with distinct different FCs between the two groups (*p* < 0.001; Table 2). The PL group had significantly higher FCs between the superior division of the right lateral occipital cortex and the right middle frontal gyrus [PL: 0.521; 95% CI (0.43–0.61); PN: 0.250 (0.14–0.36); *p* = 7.00 × 10^−4^; effect size: 1.36], the left planum temporale and the left pallidum [PL: 0.258 (0.17–0.35); PN: 0.009 (−0.08 to 0.10); *p* = 7.59 × 10^−4^; effect size: 1.35], the posterior division of the cingulate gyrus and the cerebellar posterior network [PL: 0.225 (0.13–0.32); PN; −0.024 (−0.10 to 0.06); *p* = 6.53 × 10^−4^; effect size: 1.37], and the posterior division of the cingulate gyrus and the left cerebellum crus2 [PL: 0.289 (0.20–0.38); PN: 0.078 (0.01–0.15); *p* = 9.67 × 10^−4^; effect size: 1.32] (Fig. 2a–c). The PN group had significantly higher FCs between the left frontal operculum cortex and the pars opercularis of the left inferior frontal gyrus [PL: 0.336 (0.27–0.41); PN: 0.613 (0.49–0.73); *p* = 5.11 × 10^−4^; effect size: 1.41], the left central opercular cortex and the intraparietal sulcus in the left dorsal attention networks [PL:−0.018 (−0.08 to 0.05); PN: 0.279 (0.17–0.38); *p* = 3.96 × 10^−5^; effect size: 1.74, p-FDR = 0.006], the left central opercular cortex and the left anterior supramarginal gyrus [PL: 0.039 (−0.06 to 0.14); PN: 0.325 (0.24–0.41); *p* = 1.83 × 10^−4^; effect size: 1.54, p-FDR = 0.015], and the left parietal operculum cortex and the intraparietal sulcus in the left dorsal attention networks [PL: 0.113 (0.05–0.18); PN: 0.448 (0.32–0.58); *p* = 7.38 × 10^−5^; effect size: 1.66, p-FDR = 0.012, Fig. 2d–f]. Among these FCs, the last three FCs had corrected p-values (p-FDR) less than 0.05, and either FC of the two groups was also 0.25 or more, which seemed to be a particularly meaningful relationship. As an explorative investigation to support this FDR correction result, we searched for FC in related areas.

**Table 2:** Functional connectivity analysis among 164 regions of interest.

ROI	ROI	FC; PL 16 mean (95% CI)	FC; PN 15 mean (95% CI)	*p* value (PL vs PN)	Effect size power (PL vs PN)	p-FDR
Superior division of lateral occipital cortex right	Middle frontal gyrus right	0.521 (0.43 to 0.61)	0.250 (0.14 to 0.36)	7.00 × 10^−4^	1.36 0.955	0.114
Visual lateral network right (38, −72, 13)	Superior temporal gyrus left	0.124 (0.06 to 0.19)	−0.091 (−0.18 to 0.004)	9.55 × 10^−4^	1.32 0.944	0.156
Planum temporale left	Pallidum left	0.258 (0.17 to 0.35)	0.009 (−0.08 to 0.10)	7.59 × 10^−4^	1.35 0.952	0.124
Posterior division of cingulate gyrus	Cerebellar posterior networks (0, −79, −32)	0.225 (0.13 to 0.32)	−0.024 (−0.10 to 0.06)	6.53 × 10^−4^	1.37 0.958	0.075
Posterior division of cingulate gyrus	Cerebellum crus2 left	0.289 (0.20 to 0.38)	0.078 (0.01 to 0.15)	9.67 × 10^−4^	1.32 0.944	0.075
Frontal operculum cortex left	Pars opercularis of inferior frontal gyrus left	0.336 (0.27 to 0.41)	0.613 (0.49 to 0.73)	5.11 × 10^−4^	1.41 0.966	0.083
Central opercular cortex left	Intraparietal sulcus in dorsal attention network left (−39, −43,52)	−0.018 (−0.08 to 0.05)	0.279 (0.17 to 0.38)	3.96 × 10^−5^	1.74 0.997	0.006
Central opercular cortex left	Anterior supramarginal gyrus left	0.039 (−0.06 to 0.14)	0.325 (0.24 to 0.41)	1.83 × 10^−4^	1.54 0.985	0.015
Parietal operculum cortex left	intraparietal sulcus in dorsal attention network left (−39, −43,52)	0.113 (0.05 to 0.18)	0.448 (0.32 to 0.58)	7.38 × 10^−5^	1.66 0.994	0.012
Salience supramarginal gyrus networks right (62, −35, 32)	Inferior temporal gyrus right	−0.097 (−0.18 to −0.01)	0.172 (0.09 to 0.25)	7.40 × 10^−5^	1.66 0.994	0.012
Frontoparietal lateral prefrontal cortex networks left (−43, 33, 28)	Hippocampus left	−0.186 (−0.29 to −0.09)	0.064 (−0.02 to 0.15)	9.10 × 10^−4^	1.33 0.947	0.148

Functional connectivity analysis among 164 regions of interest detected 11 connection pairs with significantly different values between the two patient groups at uncorrected *p* < 0.001. Values in the PL group and PN group were analyzed by Student’s t test. Corrected p values using the False Discovery Rate (FDR) are also written as p-FDR.

*ROI* region of interest, *FC* functional connectivity.

**Figure 3:**
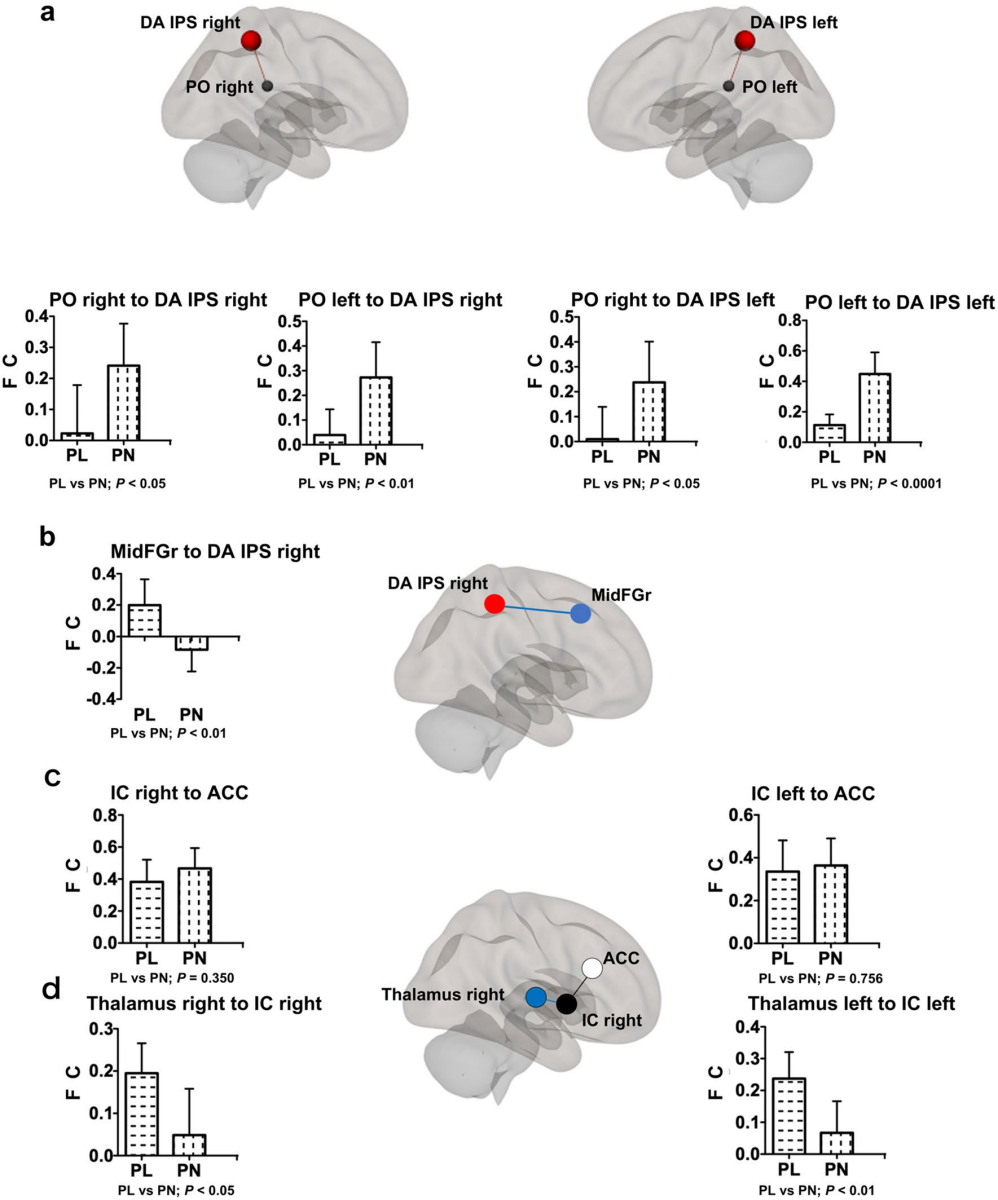
Significant differences in FC between the two groups were observed in several regions related to pain perception. (**a**) Functional connectivity of the parietal operculum cortex to the intraparietal sulcus in the dorsal attention network was significantly higher on both the same side and the contralateral side in the PN group than that in the PL group. **(b**) Functional connectivity of the middle frontal gyrus right in the ventral attention network to the right intraparietal sulcus in the dorsal attention network was significantly higher in the PL group. (**c**) Functional connectivity of the right and left insular cortex to the anterior cingular cortex was high in both patient groups, whereas there was no significant difference between them (right; *p* = 0.350, left; *p* = 0.756). (**d**) Functional connectivity between the thalamus and the insular cortex on both hemispheres was significantly higher in the PL group. Graphs represent mean and 95% CI values for each group. *FC* functional connectivity, *DA IPS* intraparietal sulcus in dorsal attention network, *PO* parietal operculum cortex, *MidFGr* middle frontal gyrus right, *IC* insular cortex, *ACC* anterior cingular cortex.

We identified a significantly higher FC of the left parietal operculum cortex, the secondary somatosensory area (S2)^28^, to the intraparietal sulcus, a major region of the dorsal attention network in the PN group (p-FDR = 0.012)^29^. Since the parietal operculum, the secondary somatosensory area, along with the posterior insular appear to be the core region for the pain perception^25^, therefore, we focused on these FCs. The FCs of the parietal operculum cortex to the intraparietal sulcus in the dorsal attention network were significantly higher on both the same side (left; *p* = 7.38 × 10^−5^; effect size: 1.66; right: *p* = 0.033; effect size: 0.81) and the contralateral side (*p* = 7.98 × 10^−3^; effect size: 1.02; *p* = 0.026; effect size: 0.85) in the PN group than those in the PL group (Fig. 3a, Supplementary Table 1). Since the PL group had significantly higher FC between the superior division of the right lateral occipital cortex and the right middle frontal gyrus (Fig. 2a), a key region of the ventral attention network^29^, we investigated interactions between the dorsal attention network and the ventral attention network. In fact, the right middle frontal gyrus has been proposed to be at the interface between the dorsal and ventral attention networks^30^. The FC of the right middle frontal gyrus in the ventral attention network to the right intraparietal sulcus in the dorsal attention network was significantly higher than that in the PL group (PL: 0.199 (0.05–0.35); PN:—0.083 (−0.21–0.04; *p* = 9.56 × 10^−3^; effect size: 1.00; Fig. 3b, Supplementary Table 1).

**Figure 4:**
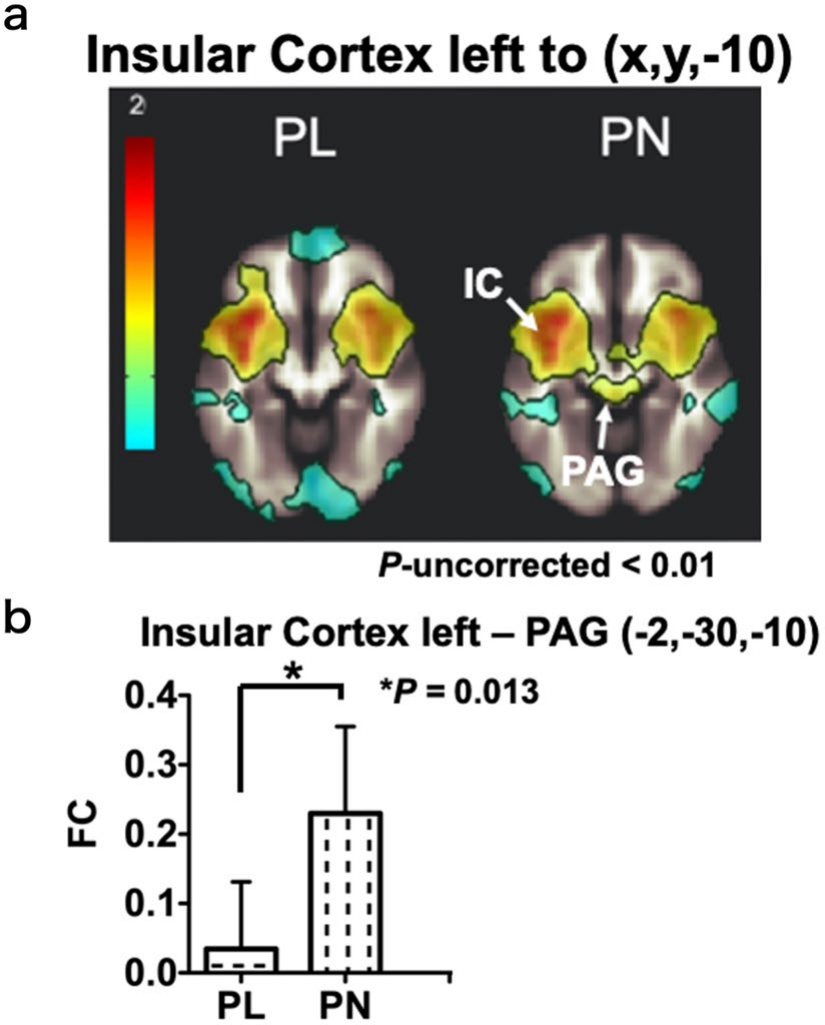
Functional connectivity of the insular cortex left to the periaqueductal gray region. **(a**) Functional connectivity to the insular cortex left in the brainstem regions (x, y, −10). Higher connectivity was observed around the periaqueductal gray region in the PN group. PAG; periaqueductal gray (white arrow; −2, −30, −10), IC; insular cortex. (**b**) Functional connectivity of the insular cortex left to the periaqueductal gray region (−2, −30, −10) in the PN group was significantly higher than that in the PL group. Graphs represent mean and 95% CI values for each group. *FC* functional connectivity, *PAG* periaqueductal gray.

Then, we investigated the transmission pathway of pain stimuli. Although FCs of the right and left insular cortex to the anterior cingular cortex were high [right: PL 0.383 (0.26–0.51); PN: 0.466 (0.35–0.58); left: PL: 0.335 (0.20–0.47); PN: 0.364 (0.25–0.48)], there was no significant difference between the two groups (right: *p* = 0.350; effect size: 0.34; left: *p* = 0.756; effect size: 0.11; Fig. 3c,d, Supplementary Table 1). On the other hand, FCs between the thalamus and the insular cortex on both hemispheres were significantly higher in the PL group [left; PL 0.237 (0.16–0.31); PN 0.067 (−0.02 to 0.16), *p* = 0.008; effect size: 1.02; right; PL 0.195 (0.13–0.26); PN 0.049 (−0.05 to 0.15), *p* = 0.021; effect size: 0.87]. The FC of the left insular cortex to the PAG region (−2, −30, −10) was significantly higher in the PN group [PL: 0.035 (−0.05 to 0.12); PN: 0.230 (0.12–0.34); *p* = 0.013: effect size: 0.96; Fig. 4; Supplementary Table 1].

## Discussion

Patients diagnosed with fibromyalgia based on the 2016 ACR criteria were assigned to the two groups based on pain threshold in this study. Patients in the PL group had a much lower pain threshold and felt even a slight touch as pain, whereas the pain threshold of patients in the PN group was equivalent to that of healthy subjects. Nevertheless, patients from both groups complained of severe widespread pain; differences in biological and pathological backgrounds may be responsible for these observations. We performed rs-fMRI analysis to investigate differences in the neural connections of these two patient groups. The most distinctive finding was that the PN group had significantly higher functional connectivity between the secondary somatosensory area and the dorsal attention network (p-FDR = 0.012). Then, we also investigated the transmission pathway of pain stimuli from the outside. Functional connectivity of the thalamus to the insular cortex was significantly higher in the PL group. The FC of the left insular cortex to the PAG region (−2, −30, −10), a part of the descending pain modulation system, was significantly higher in the PN group.

Recent neuroimaging studies showed that several areas of the brain are implicated in the painful symptoms of fibromyalgia. Patients with fibromyalgia are known to exhibit an increased FC of the insular cortex to various regions of the brain, including the DMN^31^ and the cingulate cortex^20^. The posterior insula, primary somatosensory cortex, and motor cortex have been reported to be more strongly connected to other brain regions in patients with fibromyalgia who experience the highest pain intensity^20^. Acute pressure pain is also associated with increased connectivity of the insular cortex with the anterior cingulate cortex, posterior cingulate cortex, and the hippocampus of patients with fibromyalgia^32^. Other areas of interest are the brainstem, including the PAG, as these regions relay the descending pain modulation system that suppresses nociceptive transmission in the dorsal horn of the spinal cord. Previous research has reported an increased FC of the PAG with the insular cortex, anterior cingulate cortex, and anterior prefrontal cortex in patients with fibromyalgia^26^.

The present study sheds light on the essential role of the attention networks in the pathogenesis of fibromyalgia. Two brain networks have attracted attention: the dorsal attention network and the ventral attention network^29^. The dorsal attention network selects sensory stimuli based on internal working memory or expectations (goal-driven attention) and triggers appropriate defensive responses (top-down signals). The ventral attention network receives new sensory stimuli from the external environment (stimulus-driven attention) and induces adaptive responses (bottom-up signals)^29^. These two systems interact in a dynamic manner so that signals via the ventral attention network can reorient information from the dorsal attention network to enable us to respond more appropriately to external environments^29^. Pain perception is a form of sensory information that is processed by these two attention networks^33^^,^^34^.

ROI-to-ROI analysis identified several relationships that differed significantly between the two patient groups. We identified a relationship between the parietal operculum cortex, the secondary somatosensory area (S2)^28^, and the intraparietal sulcus in the dorsal attention network; this is one of the main regions of the network^29^. The FCs between these two regions were significantly greater in the PN group (Fig. 3a, Supplementary Table 1). The insula, especially the posterior portion, and the second somatosensory area, receive sensory input from the thalamus; subsequently, these regions perceive sensory stimulation and encode pain sensation^24^^,^^25^. Painful stimuli activate several brain regions, including the primary and secondary somatosensory areas, insular cortex, anterior cingulate, prefrontal cortices, and the thalamus; collectively, these regions are known as the pain matrix^25^. However, of these, the regions in which somatic pain sensation is induced by electrical stimulation are limited only to the medial parietal operculum and the posterior insular cortex^35^^,^^36^. Thus, the medial parietal operculum and the posterior insular appear to be the core region for the perception of internal pain^25^. The PN group had significantly greater FCs of the parietal opercular cortex to the dorsal attention network (Figs. 2e, 3a). Therefore, patients in the PN group may have experienced endogenous pain due to the top-down signal driven by the dorsal attention network.

We observed an increased FC of the lateral occipital cortex, which receives visual sensations^37^, to the right middle frontal gyrus in the PL group of patients with a lower pain threshold (Fig. 2a). The ventral attention network receives external sensory stimuli from the environment and responds accordingly, leading to reorientation of the dorsal attentional network with pre-existing working memory; this occurs via activation of the right middle frontal gyrus of the ventral attentional network (Fig. 3b)^29^. FCs between the thalamus and the insular cortex were higher in the PL group than in the PN group (Fig. 3d). Thus, PL patients may feel external pain stimuli in a sensitive manner.

Patients in the PL group had significantly reduced pain thresholds. The WPI score was significantly higher than that of the PN group. In this study, pain thresholds were measured at the fingertips, but if patients in the PL group had lower pain thresholds systemically as the fingertips, the area where they felt pain might be larger. We observed an increased FC between the left insular cortex to the PAG region (−2, − 30, −10) in the PN group (Fig. 4). In the PN group, the descending pain modulation system may function in response to pain sensation recognized by the insular cortex, maintaining an appropriate pain threshold. However, in the PL group, the descending pain modulation system may be impaired, possibly resulting in a lower pain threshold.

Some patients in the PL group showed symptoms corresponding to chronic fatigue syndrome.

Although there is currently no clear evidence, patients with low pain thresholds may have some impairment or damage to their peripheral nervous system, central nervous system, or both caused by neuroinflammation, autoimmune reactions or impaired energy metabolism like Long COVID^38–40^. In addition to dysfunction of the descending pain modulation system, spinal glial activation^41^, abnormal terminal connectivity, and malfunction of nociceptors during reinnervation^42^ may possibly influence pain threshold. It seems to have properties of neuropathic pain. The efficacy of treatments targeting redox imbalance is being investigated for these pathological conditions^43^. On the other hand, patients with normal pain thresholds may experience pain due to changes in neural circuitry resulting from repeated neural transmissions that are at least initially normal^44^. It seems to have more of a nociplastic pain quality^15^. Therefore, even if there is damage to nerve tissue, it is likely to be mild and reversible. In this regard, music^45^, acupuncture^46^, and cognitive-behavioral therapy^47^ have all been reported as effective treatments for pain in some patients with fibromyalgia. These non-pharmacological therapies may exert therapeutic effects by reorienting the working memory of the dorsal attention network in certain patients with fibromyalgia such as those in the PN group. These possibilities should be considered in future studies.

There are several limitations. This study did not compare healthy subjects with patients. While many imaging studies already exist, the problem of comparing pain-free healthy subjects with painful patients is that we cannot be sure whether findings are the cause of pain sensation or the result of pain perception. Although we targeted a small number of patients, by focusing on pain threshold, we could compare two patient groups with equivalent levels of pain sensation but with different characteristics. Rs-fMRI analysis cannot necessarily clarify neuropathological causal relationships. We found that the dorsal attention network may play a key role in a subgroup of patients with fibromyalgia and that this is more likely to represent the cause rather than the result of pain perception, thus fitting the concept of nociplastic pain disorder (Supplementary Fig. 1)^15^.

In conclusion, this study suggests that endogenous pain driven by top-down signals via the dorsal attention network may contribute to pain sensation in a subgroup of fibromyalgia patients with a normal pain threshold, while another subgroup of fibromyalgia patients with a low pain threshold may feel external pain driven by bottom-up signals via the spinothalamic tract. Consequently, it would be more desirable to provide treatments that match each patient’s individual condition.

## Methods

### Study design and setting

This single center, observational, and case-controlled study included two groups of patients attending the outpatients clinic at the Pain Center (Teikyo University Chiba Medical Center, Ichihara) between October 2019 and June 2022. This study was performed in accordance with the Declaration of Helsinki and was approved by the Teikyo University Ethical Review Board for Medical and Health Research Involving Human Subjects (approval number: 19-077; 17th of July 2019). The study was prospectively registered in the University Hospital Medical Information Network Clinical Trials Registry (UMIN000037712, 26th of August 2019; Principal investigator: Tomohiko Aoe, https://center6.umin.ac.jp/cgi-open-bin/ctr/ctr_view.cgi?recptno=R000042918). All participants provided written informed consent to participate. This study adheres to the applicable STROBE Statement (https://www.strobe-statement.org).

### Participants

Patients who visited the outpatient clinic and met the 2016 ACR fibromyalgia diagnostic criteria (0–31; the widespread pain index (WPI) up to 19 + the symptom severity scale (SSS) up to 12)^5^ were eligible if they had experienced stable symptoms for three months prior to imaging. Patients were assigned to the two groups based on pain threshold, as described later. Patients were excluded if they had any of the following conditions; a history of neurological or major psychiatric illness, a significant medical disorder, alcohol/drug abuse, left handedness to eliminate the influence of hemispheric dominance, current pregnancy or breastfeeding, or if they were less than 20 years-of-age or over 70 years-of-age. Patients were also excluded if they had conditions that may have interfered with the MRI procedure either from a mental or physical context, or if they had abnormal findings on simple brain MRI. In a similar study, the sample size was set to 14–15 in each group^48^. Power calculation for an expected effect size (d) of 1.0 in the values between the two groups (d ≥ 0.8 is classified as large)^49^, with a two-tailed α probability level of 0.05 and a power of 0.80 (1 − β) yielded a sample size of 17 patients for each group. Consequently, we aimed to recruit 17 individuals in each group.

### Variables

#### Pressure pain threshold

For each patient, we measured the pressure pain threshold (PPT)^27^ with a pressure algometer (NEUTONE TAM-Z2, TRY-ALL, Chiba, Japan) equipped with a probe (diameter: 8 mm; area: 0.5 cm^2^). The evaluator applied force to the tip of the patient’s index finger and measured the value when the patient felt pain; this was repeated three times for both the right and left hands. The mean value (kg/0.5 cm^2^) was then used as the PPT value. We chose fingertips because they can be easily tested in outpatient clinics, and the tests are reproducible. A recent study indicates that most fibromyalgia patients don’t report their fingertips as painful body regions^18^. Previously, we investigated 25 women (mean age: 42.6 ± 7.3 years) with no complaints of chronic pain with a mean value of 2.44 with a standard deviation of 0.75^27^. The PPT value with 2.58Z variation (*P* < 0.01) from the mean value becomes 0.5 kg. Therefore, patients with an average PPT value of ≤ 0.5 kg were assigned to the PL group (patients with a low pain threshold) while other patients were assigned to the PN group (patients with a normal pain threshold).

#### Numeric rating scale

The numeric rating scale (NRS) evaluates the degree of pain in 11 stages ranging from 0–10, with 0 indicating no pain at all and 10 indicating the worst pain possible. In this study, the evaluator asked each patient to provide a NRS score.

#### Pain catastrophizing scale

The pain catastrophizing scale (PCS) evaluates three components of catastrophizing: rumination, magnification, and helplessness. We asked each patient thirteen questions to indicate the extent to which they had experienced thoughts and feelings related to rumination, magnification, and helplessness using a scale of 0 (never) to 4 (always). The total score was calculated on a scale ranging from 0 to 52. A higher PCS score represented a level clinically equivalent to catastrophic in chronic pain patients.

#### Resting-state fMRI

MRI data were acquired with a 3.0 Tesla MRI scanner (GE Healthcare, Discovery MR750, Milwaukee, WI, USA) with a head, neck, and spine array coil (GE Healthcare). The patient's head was secured with a soft pad to reduce movement during scanning. Patients were instructed to close their eyes while remaining awake. Alertness was confirmed by asking each patient to respond to questions before and after the scan. rs-fMRI images of 140 brain volumes were obtained from a 5 min scan using a blood oxygen level-dependent gradient echo-planar pulse sequence (repetition time: TR = 2000 ms; echo time: TE = 30 ms; voxel size: 3.44 × 3.44 × 3.5 mm^3^; flip angle: 90°; number of slices: 40). We also acquired T1-weighted images (voxel size: 0.86 × 0.86 ×   1.0 mm^3^). T1-weighted images were spatially normalized to the Montreal Neurological Institute (MNI) template^50^. Then, we performed data pre-processing after removing the initial six volumes, using Statistical Parametric Mapping (SPM12, Wellcome Centre for Human Neuroimaging, London, UK) and CONN Toolbox 2019 (McGovern Institute for Brain Research, MIT, Cambridge, MA, USA)^51^ running on MATLAB R2020b (MathWorks, Natick, MA, USA). CONN’s default MNI pipeline consists of the following steps: functional realignment and unwarping with subject motion estimation and correction, functional and structural centering to (0, 0, 0) coordinates, slice-timing correction, structural segmentation and normalization, functional normalization, outlier detection, and smoothing. Then, we applied a denoising step for linear regression and band-pass filtering to remove unwanted motion, physiological, and other artifactual effects from the blood oxygen-dependent signal before calculating connectivity indices. The default CONN preprocessing steps were automatically generated to use a combination of anatomical component correction, scrubbing, motion regression, and filtering in the denoising step. Then, we obtained ROI-to-ROI and ROI-to-voxel FCs. CONN calculates ROI-to-ROI connectivity based on the combination of anatomical and functional parcellation using a total of 164 areas, 32 areas of functional network and 132 areas of anatomical atlas. The anatomical parcellation composed of 91 cortical ROIs and 15 subcortical ROIs based on the Harvard–Oxford atlas (https://fsl.fmrib.ox.ac.uk/fsl/fslwiki/Atlases), and 26 cerebellar areas from the Automated Anatomical Labeling atlas^52^. The functional parcellation composed of eight networks (Cerebellar, Fronto Parietal, Default Mode, SensoriMotor, Dorsal Attention, Language, Salience, and Visual) represented by 32 regions (Supplementary Methods).

### Statistical analysis

Data are expressed as mean values with ± SD or 95% CIs. Two-tailed Student’s t tests were used to compare data between the two patient groups. Data analysis was performed with Prism 4.0 (GraphPad Software, San Diego, CA, USA), Microsoft Excel software (Microsoft Corporation, Redmond, WA, USA). *p* < 0.05 was considered to be statistically significant. Effect sizes were calculated from the obtained results, and statistical power at *p* < 0.05 was calculated by post hock analysis. Sample size estimation and power analysis were performed by G*Power 3 (Faul, Erdfelder, Lang & Buchner, 2007: http://www.psycho.uni-duesseldorf.de/abteilungen/aap/gpower3). P-values related to FC are written as uncorrected p-values, and those corrected using the False Discovery Rate (FDR) are written as p-FDR.

### Supplementary Information

Supplementary Information.

## Data Availability

The datasets generated during and/or analysed during the current study are available from the corresponding author on reasonable request.
