# Supplementary material for: Functional connectivity associated with attention networks differs among subgroups of fibromyalgia patients: an observational case–control study
Source: Sci Rep. 2024 May 3;14:10197. doi: 10.1038/s41598-024-60993-9 (PMC11068894; doi:10.1038/s41598-024-60993-9)
Supplement: Supplementary file 1 — Supplementary Information. [file 41598_2024_60993_MOESM1_ESM.pdf]

Supplementary Information

Functional connectivity associated with attention networks differs among subgroups of fibromyalgia patients: an observational case-control study

Tomohiko Aoe, Ryoko Kawanaka, Fumio Ohsone, Akira Hara, Tokuzo Yokokawa

Supplementary Figure 1

Possible hypothesis

Supplementary Table 1

Functional connectivity between several regions of interest with regards to pain perception and attention.

Supplementary Methods

Methods for resting-state fMRI analysis of this study

Supplementary Table 1

Functional connectivity between several regions of interest with regards to pain perception and attention

ROI	ROI	FC; PL 16 mean (95% CI)	FC; PN 15 mean (95% CI)	*p* value (PL vs PN)	effect size power (PL vs PN)
parietal operculum cortex left	intraparietal sulcus in dorsal attention networks. left (-39,-43,52)	0.113 (0.05-0.18)	0.448 (0.32-0.58)	7.38×10^{-5}	1.66 0.994
parietal operculum cortex left	intraparietal sulcus in dorsal attention networks. right (39,-43,52)	0.039 (-0.06-0.13)	0.273 (0.14-0.40)	7.98×10^{-3}	1.02 0.783
parietal operculum cortex right	intraparietal sulcus in dorsal attention networks. left (-39,-43,52)	0.010 (-0.11-0.13)	0.238 (0.09-0.39)	0.026	0.85 0.628
parietal operculum cortex right	intraparietal sulcus in dorsal attention networks. right (39,-43,52)	0.023 (-0.12-0.17)	0.241 (0.12-0.36)	0.033	0.81 0.587
middle frontal gyrus right	intraparietal sulcus in dorsal attention networks. right (39,-43,52)	0.199 (0.05-0.35)	-0.083 (-0.21-0.04)	9.56×10^{-3}	1.00 0.767
thalamus left	insular cortex left	0.237 (0.16-0.31)	0.067 (-0.02-0.16)	0.008	1.02 0.783
thalamus right	insular cortex right	0.195 (0.13-0.26)	0.049 (-0.05-0.15)	0.021	0.87 0.648
insular cortex left	anterior cingular cortex	0.335 (0.20-0.47)	0.364 (0.25-0.48)	0.756	0.11 0.06
insular cortex right	anterior cingular cortex	0.383 (0.26-0.51)	0.466 (0.35-0.58)	0.350	0.34 0.15
insular cortex left	periaqueductal gray left (-2,-30,-10)	0.035 (-0.05-0.12)	0.230 (0.12-0.34)	0.013	0.96 0.733

Values in the PL group and PN group were analyzed by Student’s t test. p values are uncorrected.

ROI; region of interest, FC; functional connectivity

Supplementary Figure 1

Possible hypothesis

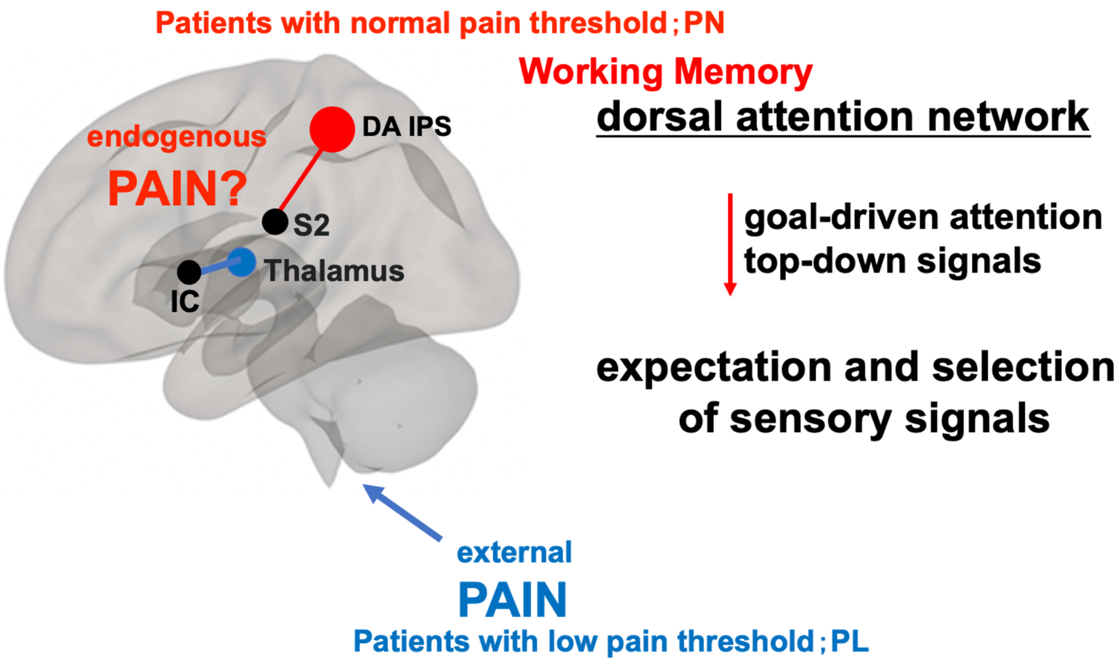

A subgroup of fibromyalgia patients with a normal pain threshold may feel endogenous pain driven by top-down signals *via* the dorsal attention network, while another subgroup of fibromyalgia patients with a low pain threshold may feel external pain driven by bottom-up signals *via* the spinothalamic tract.

DA IPS; intraparietal sulcus in dorsal attention network, IC; insular cortex, S2; the secondary somatosensory area

Supplementary Methods

1. This text is distributed under a Public Domain Dedication license (CC0 1.0) and provided by CONN software for this study.

Methods

Results included in this manuscript come from analyses performed using CONN[1] (RRID:SCR_009550) release 19.c[2] and SPM[3] (RRID:SCR_007037) release 12.7487.

Preprocessing: Functional and anatomical data were preprocessed using a flexible preprocessing pipeline[4] including removal of initial scans, realignment with correction of susceptibility distortion interactions, slice timing correction, outlier detection, direct segmentation and MNI-space normalization, and smoothing. The first 6 scans in each functional run were removed. Functional data were realigned using SPM realign & unwarp procedure[5], where all scans were coregistered to a reference image (first scan of the first session) using a least squares approach and a 6 parameter (rigid body) transformation[6], and resampled using b-spline interpolation to correct for motion and magnetic susceptibility interactions. Temporal misalignment between different slices of the functional data (acquired in ascending order) was corrected following SPM slice-timing correction (STC) procedure[7,8], using sinc temporal interpolation to resample each slice BOLD timeseries to a common mid-acquisition time. Potential outlier scans were identified using ART[9] as acquisitions with framewise displacement above 0.9 mm or global BOLD signal changes above 5 standard deviations[10,11], and a reference BOLD image was computed for each subject by averaging all scans excluding outliers. Functional and anatomical data were normalized into standard MNI space, segmented into grey matter, white matter, and CSF tissue classes, and resampled to 2 mm isotropic voxels following a direct normalization procedure[11,12] using SPM unified segmentation and normalization algorithm[13,14] with the default IXI-549 tissue probability map template. Last, functional data were smoothed using spatial convolution with a Gaussian kernel of 8 mm full width half maximum (FWHM).

Denoising: In addition, functional data were denoised using a standard denoising pipeline[15] including the regression of potential confounding effects characterized by white matter timeseries (5 CompCor noise components), CSF timeseries (5 CompCor noise components), motion

parameters and their first order derivatives (12 factors)[16], outlier scans (below 5 factors)[10], session effects and their first order derivatives (2 factors), and linear trends (2 factors) within each functional run, followed by bandpass frequency filtering of the BOLD timeseries[17] between 0.008 Hz and 0.09 Hz. CompCor[18,19] noise components within white matter and CSF were estimated by computing the average BOLD signal as well as the largest principal components orthogonal to the BOLD average, motion parameters, and outlier scans within each subject's eroded segmentation masks.

First-level analysis SBC_01: Seed-based connectivity maps (SBC) and ROI-to-ROI connectivity matrices (RRC) were estimated characterizing the patterns of functional connectivity with 164 HPC-ICA networks[2] and Harvard-Oxford atlas ROIs[20]. Functional connectivity strength was represented by Fisher-transformed bivariate correlation coefficients from a weighted general linear model (weighted-GLM[21]), defined separately for each pair of seed and target areas, modeling the association between their BOLD signal timeseries.

Group-level analyses were performed using a General Linear Model (GLM[22]). For each individual voxel a separate GLM was estimated, with first-level connectivity measures at this voxel as dependent variables (one independent sample per subject and one measurement per task or experimental condition, if applicable), and groups or other subject-level identifiers as independent variables. Voxel-level hypotheses were evaluated using multivariate parametric statistics with random-effects across subjects and sample covariance estimation across multiple measurements. Inferences were performed at the level of individual clusters (groups of contiguous voxels). Cluster-level inferences were based on parametric statistics from Gaussian Random Field theory[23,24]. Results were thresholded using a combination of a cluster-forming $p < 0.001$ voxel-level threshold, and a familywise corrected p-FDR < 0.05 cluster-size threshold[25].

References

[1] Whitfield-Gabrieli, S., & Nieto-Castanon, A. (2012). Conn: a functional connectivity toolbox for correlated and anticorrelated brain networks. Brain connectivity, 2(3), 125-141.

[2] Nieto-Castanon, A. & Whitfield-Gabrieli, S. (2019). CONN functional connectivity toolbox: RRID SCR_009550, release 19. doi:10.56441/hilbertpress.1927.9364.

[3] Penny, W. D., Friston, K. J., Ashburner, J. T., Kiebel, S. J., & Nichols, T. E. (Eds.). (2011). Statistical parametric mapping: the analysis of functional brain images. Elsevier.

[4] Nieto-Castanon, A. (2020). FMRI minimal preprocessing pipeline. In Handbook of functional connectivity Magnetic Resonance Imaging methods in CONN (pp. 3–16). Hilbert Press.

[5] Andersson, J. L., Hutton, C., Ashburner, J., Turner, R., & Friston, K. J. (2001). Modeling geometric deformations in EPI time series. Neuroimage, 13(5), 903-919.

[6] Friston, K. J., Ashburner, J., Frith, C. D., Poline, J. B., Heather, J. D., & Frackowiak, R. S. (1995). Spatial registration and normalization of images. Human brain mapping, 3(3), 165-189.

[7] Henson, R. N. A., Buechel, C., Josephs, O., & Friston, K. J. (1999). The slice-timing problem in event-related fMRI. NeuroImage, 9, 125.

[8] Sladky, R., Friston, K. J., Tröstl, J., Cunnington, R., Moser, E., & Windischberger, C. (2011). Slice-timing effects and their correction in functional MRI. Neuroimage, 58(2), 588-594.

[9] Whitfield-Gabrieli, S., Nieto-Castanon, A., & Ghosh, S. (2011). Artifact detection tools (ART). Cambridge, MA. Release Version, 7(19), 11.

[10] Power, J. D., Mitra, A., Laumann, T. O., Snyder, A. Z., Schlaggar, B. L., & Petersen, S. E. (2014). Methods to detect, characterize, and remove motion artifact in resting state fMRI. Neuroimage, 84, 320-341.

[11] Nieto-Castanon, A. (submitted). Preparing fMRI Data for Statistical Analysis. In M. Filippi (Ed.). fMRI techniques and protocols. Springer. doi:10.48550/arXiv.2210.13564

[12] Calhoun, V.D., Wager, T.D., Krishnan, A., Rosch, K.S., Seymour, K.E., Nebel, M.B., Mostofsky, S.H., Nyalakanai, P. and Kiehl, K. (2017). The impact of T1 versus EPI spatial normalization templates for fMRI data analyses (Vol. 38, No. 11, pp. 5331-5342).

[13] Ashburner, J., & Friston, K. J. (2005). Unified segmentation. Neuroimage, 26(3), 839-851.

[14] Ashburner, J. (2007). A fast diffeomorphic image registration algorithm. Neuroimage, 38(1), 95-113.

[15] Nieto-Castanon, A. (2020). FMRI denoising pipeline. In Handbook of functional connectivity Magnetic Resonance Imaging methods in CONN (pp. 17–25). Hilbert Press.

[16] Friston, K. J., Williams, S., Howard, R., Frackowiak, R. S., & Turner, R. (1996). Movement-related effects in fMRI time-series. Magnetic resonance in medicine, 35(3), 346-355.

[17] Hallquist, M. N., Hwang, K., & Luna, B. (2013). The nuisance of nuisance regression: spectral misspecification in a common approach to resting-state fMRI preprocessing reintroduces noise and obscures functional connectivity. Neuroimage, 82, 208-225.

[18] Behzadi, Y., Restom, K., Liau, J., & Liu, T. T. (2007). A component based noise correction method (CompCor) for BOLD and perfusion based fMRI. Neuroimage, 37(1), 90-101.

[19] Chai, X. J., Nieto-Castanon, A., Ongur, D., & Whitfield-Gabrieli, S. (2012). Anticorrelations in resting state networks without global signal regression. Neuroimage, 59(2), 1420-1428.

[20] Desikan R.S., Ségonne F., Fischl B., Quinn B.T., Dickerson B.C., Blacker D., Buckner R.L., Dale A.M., Maguire R.P., Hyman B.T., Albert M.S., & Killiany R.J. (2006) An automated labeling system for subdividing the human cerebral cortex on MRI scans into gyral based regions of interest. Neuroimage 31(3):968-980

[21] Nieto-Castanon, A. (2020). Functional Connectivity measures. In Handbook of functional connectivity Magnetic Resonance Imaging methods in CONN (pp. 26–62). Hilbert Press.

[22] Nieto-Castanon, A. (2020). General Linear Model. In Handbook of functional connectivity Magnetic Resonance Imaging methods in CONN (pp. 63–82). Hilbert Press.

[23] Worsley, K. J., Marrett, S., Neelin, P., Vandal, A. C., Friston, K. J., & Evans, A. C. (1996). A unified statistical approach for determining significant signals in images of cerebral activation. Human brain mapping, 4(1), 58-73.

[24] Nieto-Castanon, A. (2020). Cluster-level inferences. In Handbook of functional connectivity Magnetic Resonance Imaging methods in CONN (pp. 83–104). Hilbert Press.

[25] Chumbley, J., Worsley, K., Flandin, G., & Friston, K. (2010). Topological FDR for neuroimaging. Neuroimage, 49(4), 3057-3064.

2. Additional explanation regarding ROI

(https://link.hilbertpress.org/files/Nieto-Castanon2019.pdf)

ROI definitions. A series of files defining seeds of interest. ROIs can be defined from mask images, text files defining a list of MNI positions, or multiple-label images. The toolbox also provides a series of default pre-defined regions of interest that that will be loaded automatically. These include a series of seed areas useful for investigating resting state connectivity –regions characterizing DMN, dorsal attention network, executive control network, etc.-, as well as a complete brain parcellation including 91 cortical areas and 15 subcortical areas from the FSL Harvard-Oxford Atlas as well as 26 cerebellar areas from the AAL atlas. See the conn/*utils/otherrois/* folder for additional/optional ROI files, including Brodmann areas, large-voxel parcellations, etc.

8 networks / 32 ROIs

Cerebellar;

networks.Cerebellar.Anterior (0,-63,-30)

networks.Cerebellar.Posterior (0,-79,-32)

Fronto Parietal;

networks.FrontoParietal.LPFC (L) (-43,33,28)

networks.FrontoParietal.PPC (L) (-46,-58,49)

networks.FrontoParietal.LPFC (R) (41,38,30)

networks.FrontoParietal.PPC (R) (52,-52,45)

Default Mode;

networks.DefaultMode.MPFC (1,55,-3)

networks.DefaultMode.LP (L) (-39,-77,33)

networks.DefaultMode.LP (R) (47,-67,29)

networks.DefaultMode.PCC (1,-61,38)

SensoriMotor;

networks.SensoriMotor.Lateral (L) (-55,-12,29)

networks.SensoriMotor.Lateral (R) (56,-10,29)

networks.SensoriMotor.Superior (0,-31,67)

Dorsal Attention;

networks.DorsalAttention.FEF (L) (-27,-9,64)

networks.DorsalAttention.FEF (R) (30,-6,64)

networks.DorsalAttention.IPS (L) (-39,-43,52)

networks.DorsalAttention.IPS (R) (39,-42,54)

Language;

networks.Language.IFG (L) (-51,26,2)

networks.Language.IFG (R) (54,28,1)

networks.Language.pSTG (L) (-57,-47,15)

networks.Language.pSTG (R) (59,-42,13)

Salience;

networks.Salience.ACC (0,22,35)

networks.Salience.AInsula (L) (-44,13,1)

networks.Salience.AInsula (R) (47,14,0)

networks.Salience.RPFC (L) (-32,45,27)

networks.Salience.RPFC (R) (32,46,27)

networks.Salience.SMG (L) (-60,-39,31)

networks.Salience.SMG (R) (62,-35,32)

Visual;

networks.Visual.Medial (2,-79,12)

networks.Visual.Occipital (0,-93,-4)

networks.Visual.Lateral (L) (-37,-79,10)

networks.Visual.Lateral (R) (38,-72,13)
